# Specification and Evaluation of Plasticizer Migration Simulants for Human Blood Products: A Delphi Study

**DOI:** 10.3390/biom11081081

**Published:** 2021-07-22

**Authors:** Aurélie Thelliez, Grégory Hénard, Bruno Delorme, Sonia Chatellier, Cécile Danel, Laurent Ducoroy, Annabelle Dupont, Delphine Garrigue, Stéphanie Genay, Jean-François Goossens, Laurence Goossens, Coralie Havet, Jean-Daniel Hecq, Caroline Maeght, Isabelle Mendel, Tomé Najdovski, Pascal Odou, Guillaume Saint-Lorant, Alexandre Ung, Marie Lecoeur, Bertrand Décaudin

**Affiliations:** 1 Univ. Lille, CHU Lille, ULR 7365 GRITA—Groupe de Recherche sur les Formes Injectables et les Technologies Associées, F-59000 Lille, France; aurelie.thelliez.etu@univ-lille.fr (A.T.); cecile.danel@univ-lille.fr (C.D.); stephanie.genay@univ-lille.fr (S.G.); jean-francois.goossens@univ-lille.fr (J.-F.G.); laurence.goossens@univ-lille.fr (L.G.); pascal.odou@univ-lille.fr (P.O.); 2 Macopharma, 200 Chaussée Fernand Forest, F-59200 Tourcoing, France; gregory.henard@macopharma.com (G.H.); bruno.delorme@macopharma.com (B.D.); laurent.ducoroy@macopharma.com (L.D.); coralie.havet@macopharma.com (C.H.); caroline.maeght@macopharma.com (C.M.); 3 KREADYS, 7000 Mons, Belgium; chatels1@orange.fr; 4 CHU Lille, Service D’hémostase et Transfusion, Centre de Biologie Pathologie, F-59037 Lille, France; annabelle.dupont@univ-lille.fr (A.D.); alexandre.ung@chru-lille.fr (A.U.); 5 CHU Lille, Department of Anesthesiology and Critical Care, F-59000 Lille, France; Delphine.garrigue@chru-lille.fr; 6 CHU UCL, Drug Stability Research Group, Namur, 5530 Yvoir, Belgium; jean-daniel.hecq@skynet.be; 7 CHU Lille, Institut D’hémato-Transfusion, Unité D’hémovigilance, F-59000 Lille, France; isabelle.mendel@chru-lille.fr; 8 Service du Sang, Croix-Rouge de Belgique, rue du Fond de Maréchal, 8/1, 5020 Suarlée, Belgium; tome.najdovski@croix-rouge.be; 9 Université de Caen Normandie, Unirouen, ABTE, F-14000 Caen, France; guillaume.saint-lorant@unicaen.fr

**Keywords:** labile blood product, migration, plasticizer, simulant

## Abstract

Potentially toxic plasticizers are commonly added to polyvinyl chloride medical devices for transfusion in order to improve their flexibility and workability. As the plasticizers are not chemically bonded to the PVC, they can be released into labile blood products (LBPs) during storage. Ideally, LBPs would be used in laboratory studies of plasticizer migration from the medical device. However, short supply (i.e., limited stocks of human blood in collection centres) has prompted the development of specific simulants for each type of LBP in the evaluation of new transfusion devices. We performed a Delphi study with a multidisciplinary panel of 24 experts. In the first (qualitative) phase, the panel developed consensus definitions of the specification criteria to be met by each migration simulant. Next, we reviewed the literature on techniques for simulating the migration of plasticizers into LBPs. A questionnaire was elaborated and sent out to the experts, and the replies were synthesized in order to obtain a consensus. The qualitative study established specifications for each biological matrix (whole blood, red blood cell concentrate, plasma, and platelet concentrate) and defined the criteria required for a suitable LBP simulant. Ten criteria were suggested: physical and chemical characteristics, opacity, form, stability, composition, ability to mimic a particular clinical situation, ease and safety of use, a simulant–plastic interaction correlated with blood, and compatibility with analytical methods. The questionnaire data revealed a consensus on the use of natural products (such as pig’s blood) to mimic the four LBPs. Opinions diverged with regard to synthetic products. However, an isotonic solution and a rheological property modifier were considered to be of value in the design of synthetic simulants. Consensus reached by the Delphi group could be used as a database for the development of simulants used to assess the migration of plasticizers from PVC bags into LBPs.

## 1. Introduction

The polymer polyvinyl chloride (PVC) is widely used in the manufacture of medical devices and, more specifically, in blood bags for the collection, preparation, and storage of labile blood products (LBPs). PVC’s flexibility is increased by the addition of plasticizers. However, given that the plasticizers are not chemically bonded to the PVC, they can migrate into the LBPs [1] and thus come into contact with patients during blood transfusions. At present, the most widely used plasticizer is di-2-ethylhexyphthalate (DEHP) [2]. This compound has been classified as toxic for reproduction (category 1B), according to the European Union’s regulation EC 1272/2008 on the classification, labelling and packaging of substances and mixtures. Hence, the use of DEHP has been called into question by various national and European authorities, particularly with regard to medical devices made available in pediatric departments, neonatal units and maternity clinics. Thus, the European directive 2007/47/EC stipulates that any carcinogenic, mutagenic, or toxic to reproduction (CMR) substance in category 1A or 1B may only be used with justification if the device is intended to be used in at-risk populations. Regulation (EU) 2017/745 imposes specific requirements on the design and manufacture of medical devices with regard to the presence of certain dangerous substances. Accordingly, certain devices must not contain CMR (category 1A and 1B) compounds or endocrine disrupters above a concentration of 0.1%. This is prompting manufacturers to replace DEHP with alternative plasticizers that are supposedly less risky for patients. Human blood is very useful for assessing the migration of plasticizers from blood bags. However, low stocks of human blood in collection centres limits the availability of human blood for research and development purposes. Furthermore, there are no guidelines on standard simulants to be used in migration testing. Lastly, evaluations of new transfusion medical devices have to consider specific simulants for each type of LBP.

The objectives of the present Delphi study were therefore to (i) draw up specification criteria for plasticizer migration simulators specifically for each LBP, (ii) rank natural or synthetic products cited in the literature and/or suggested by members of an expert panel, and thus (iii) build a reliable consensus of opinion in the field of transfusion.

## 2. Materials and Methods

### 2.1. The Delphi Method

**Figure 1:**
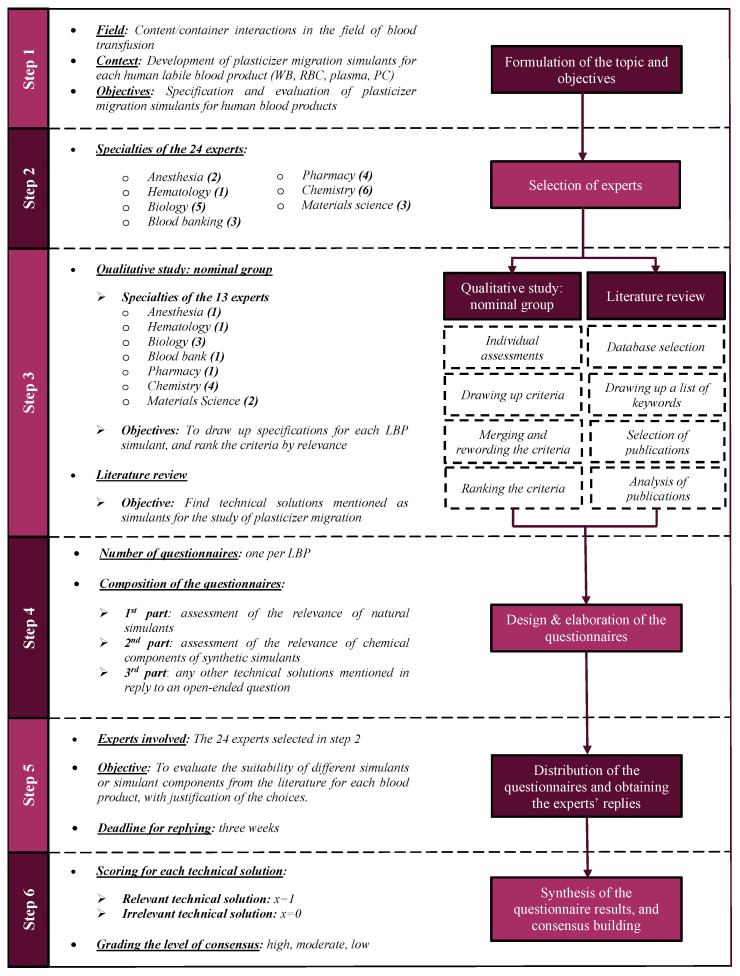
The Delphi method. LBP: labile blood product; WB: whole blood; RBC: red blood cell concentrate; P: plasma; PC: platelet concentrate.

The Delphi method is based on structured communication between several experts (Figure 1) [3,4]. The objective is to highlight convergences of opinion that enable a consensus to be reached on well-defined subjects. Questionnaires are sent individually to the experts in order to maintain the participants’ anonymity and ensure that replies are independent. The Delphi method has many advantages: a remote collection of opinions, limitation of the number of participants, a flexible methodology, implementation of successive questionnaires (allowing a consensus to be reached), and facilitated feedback during iterations.

### 2.2. Study Design

#### 2.2.1. Formulation of the Topic and the Working Group’s Objectives, and Selection of the Experts

After having explained the study’s context and objectives to potential study participants (Figure 1, step 1), we formed a multidisciplinary panel of individuals with expertise in all the required specialities: pharmacists, chemists and materials scientists for the “container” aspect, and haematologists, anaesthetists, blood bank experts (from the French Blood Establishment and the Belgian Red Cross), and biological engineers for the “content” aspect. Twenty-four experts agreed to join the panel (Figure 1, step 2) whose degrees, functions, fields of expertise, and experience are mentioned in the Supplementary Materials Section.

#### 2.2.2. Qualitative Study

We next set up a “nominal group” qualitative study (Figure 1, step 3), the objective of which was to reach a consensus on the criteria that must be met by a simulant in order to mimic plasticizer migration into human whole blood (WB), red blood cell concentrate (RBC), platelet concentrate (PC), and plasma (P) [4,5]. The multi-step nominal group method makes it possible to reach a consensus on a specific theme during a half-day, face-to-face meeting. Here, 13 of the 24 Delphi experts participated in the meeting. In the first stage, each expert considers the questions individually and formulates possible answers. Next, each expert chooses criteria from among those described on his/her preparatory document. Several rounds were therefore held in order to exhaust all the criteria selected by the experts. In the third step, similar criteria are merged, reformulated, and clarified. Lastly, the criteria characterizing each LBP were ranked in order of importance (from a value of 1 for the most important criterion up to a value of n for the least important criterion, where n = the number of criteria) by each expert of the nominal group.

#### 2.2.3. Literature Review

The literature review was carried out between March and May 2020, in parallel with the qualitative study. The goal was to find technical solutions for use as simulants in plasticizer migration studies (Figure 1, step 3). By applying five sets of keywords in English (Table 1), we searched the PubMed database for full-text articles published in English or French. No limitation was placed on the search period (i.e., all fields and no filters). The sets of keywords were used separately first but were then combined in order to reduce the number of publications and focus the search on the topic in question. The following combinations were used: 1-2-3-4-5, 1-3-4-5, and 2-3-4-5. All results were reviewed, selected, and validated by the study’s scientific advisory board. Lastly, we screened the bibliographies of all the selected full-text publications for additional studies of human blood simulants.

#### 2.2.4. Design and Elaboration of the Questionnaires

All the criteria defined during the nominal group meeting and all the technical solutions extracted from the literature search were used to draw up the questionnaire (Figure 1, step 4). A three-part matrix questionnaire was built: the first part assessed the relevance of naturally derived simulants, the second assessed relevance of chemically derived components for synthetic simulants, and the third contained an open-ended question that enabled the experts to cite technical solutions that had not been mentioned previously. The questionnaires were tested in a pilot study, in order to identify ambiguities and improve the phrasing, if required. The questionnaire was designed using Google Forms.

#### 2.2.5. Distribution of the Questionnaires

All 24 experts in the Delphi group participated in the questionnaire step (Figure 1, step 5). For each human blood product, each expert had to evaluate the relevance of various simulants or components of simulants from the literature and justify their choice. A link to the questionnaire was sent to the experts by e-mail. The experts were given a deadline of three weeks to send their reply.

#### 2.2.6. Synthesis of the Questionnaire Results and Consensus Building

Each technical solution was assigned a value of x = 1 when it was judged to be relevant and x = 0 when it was judged to be irrelevant (Figure 1, step 6) [6]. The overall relevance score was calculated as follows:

Score (%) = (Σx = 1)/(Number of experts) × 100(1)

The answers were not weighted with regard to the respondent’s field of expertise. When a participant did not feel knowledgeable enough to answer a question, he/she could request that the answer be discarded. Likewise, any answers that were not justified by the respondent were excluded from the final results. A consensus scale (Table 2) was set up to assess the degree of consensus reached on each technical solution. Higher scores and lower scores corresponded to a greater degree of consensus on the solution’s relevance or irrelevance, respectively. A score of between 35% and 65% corresponded to a lack of consensus.

## 3. Results and Discussion

### 3.1. The Qualitative Study

Firstly, the nominal group meeting’s topic, objectives, and methodology were presented to the 13 participating experts. After each person had considered the issues individually, the experts suggested several criteria for characterization of the most suitable simulants in plasticizer migration studies. In total, 52, 46, 42, and 48 criteria were suggested for WB, RBC, plasma, and PC, respectively. Although the criteria were formulated differently, it was possible to group, rephrase, and/or delete some criteria by consensus; this reduced the number of criteria and facilitated their ranking. Hence, there were 10 reformulated criteria, each for WB and RBC, and nine each for plasma and PC (Table 3).

**Figure 2:**
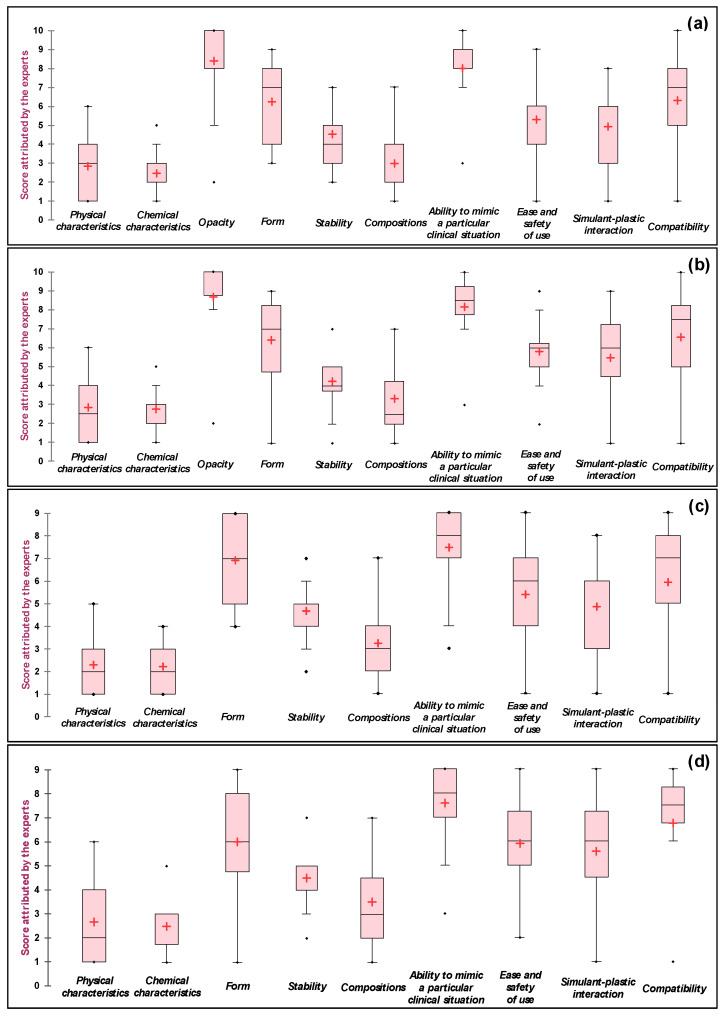
Box plots of the scores obtained for each criterion related to each labile blood product, as attributed by the members of the nominal group. (**a**) Whole blood, (**b**) red blood cell concentrate, (**c**) plasma, and (**d**) platelet concentrate. +: mean value; ◆: outlier value.

The reformulated criteria characterizing each LBP were then scored for relevance (with a value of 1 for the most relevant criterion, up to 9 or 10 (depending on the LBP) for the most irrelevant criterion) by each expert in the nominal group. The relevance scores for each LBP were represented as box plots (Figure 2). Whatever the LBP, it can be noted that all the experts considered the “chemical characteristics” criterion to be relevant (median value: 2–3) and the “capacity to mimic a clinical situation” criterion to be less relevant (median value = 8–9). The degree of dispersion was low, i.e., the scores’ interquartile ranges were narrow. In contrast, the participants disagreed on the relevance of the “form”, “composition”, “ease and safety of use”, and “simulant-plastic interaction” criteria, for which the scores’ ranges were large (e.g., a range of between 7 and 8 for the “simulant-plastic interaction” criterion). However, by referring to the values of the means and medians, we were able to establish a three-level classification (Table 4) for each LBP: relevant (median ≤ 3), moderately relevant (3 < median ≤ 6), and less relevant (median > 6).

Although the score obtained for each criterion differed according to the expert’s speciality, it was possible to establish an order of importance by summing the ranking scores given by each member of the nominal group for each LBP (Table 5). It was found that the ranking of the criteria is identical for WB and RBC. For plasma, an inversion between the “form” and “compatibility” criteria ranking compared to RBC and WB was observed as well as an absence of the “opacity” criterion, which was not selected for this LBP. Except for the opacity factor, which was not assessed, the specifications for PC are similar to those for WB and RBC.

### 3.2. Review of the Literature and Elaboration of Questionnaires

**Figure 3:**
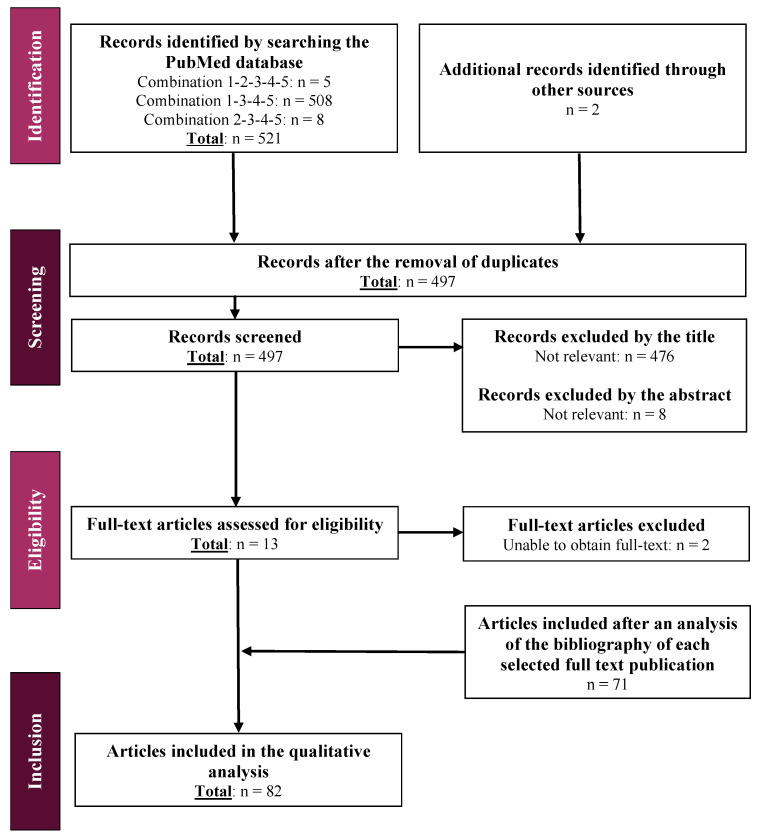
Flow chart for the literature search and review.

The literature review was performed by combining the keywords defined in Table 1. A total of 497 publications were screened successively by title, abstract, and full text: 82 articles met all the acceptance criteria (Figure 3).

The literature review enabled us to identify several technical solutions for simulating human blood in studies of the migration of plasticizers from medical devices [7,8,9,10,11,12,13,14,15,16,17,18,19,20,21,22,23,24,25,26,27,28,29,30,31,32,33,34,35,36,37,38,39,40,41,42,43,44,45,46,47,48,49,50,51,52,53,54,55,56,57,58,59,60,61,62,63,64,65,66,67,68,69,70,71,72,73,74,75,76,77,78,79,80,81,82,83,84,85,86,87,88,89]. The solutions were classified as natural *versus* synthetic products (Table 6). The study questionnaire, therefore, covered the criteria that must be met by a simulant (as proposed by the nominal group) and the technical solutions extracted from the literature review.

### 3.3. Questionnaire Results and the Consensus Reached

**Figure 4:**
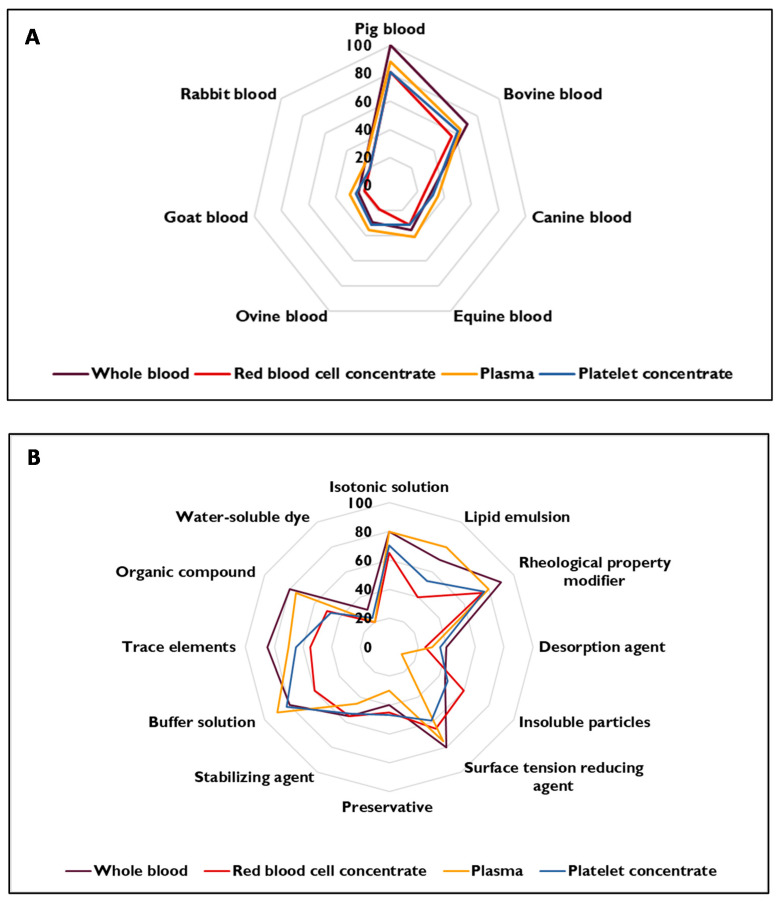
Relevance scores for each natural technical solution (**A**) or synthetic technical solution (**B**) for each labile blood product.

After analyzing the questionnaires, we calculated the relevance scores (in %) for each technical solution (natural or synthetic) and each LBP (Figure 4). The degree of consensus was then noted, according to the scale shown in Table 2. The experts’ comments highlighted differences of opinion with regard to the design of a synthetic simulant for LBPs. Indeed, some experts considered that a synthetic simulant could not adequately mimic the migration of plasticizers into an LBP relative to a natural simulant. However, other experts considered that synthetic products were safer, more reproducible, and potentially available in larger quantities.

#### 3.3.1. Technical Solutions of Natural Origin

##### Technical Solutions of Natural Origin for Whole Blood

For WB, a high level of consensus was found for pig’s blood: all the experts agreed that this was a relevant technical solution for mimicking the migration of plasticizers into human WB. Bovine WB was also considered to be a relevant technical solution, albeit with a lower level of consensus (71%). The five other points of consensus attested to the irrelevance of canine, equine, ovine, goat, and rabbit WBs for mimicking the migration of plasticizers into human WB. Indeed, goat, and rabbit WBs had a moderate degree of consensus, and canine, equine, and ovine WBs had a low level of consensus.

##### Technical Solutions of Natural Origin for Red Blood Cell Concentrate

A moderate degree of consensus was found for pig RBC since 81% of the experts agreed that this technical solution was suitable for mimicking the migration of plasticizers into human RBCs. Regarding bovine RBCs, no consensus was observed because the experts’ opinions were divided (between 35% and 65%). With a moderate degree of consensus for canine, ovine, goat, and rabbit RBCs and a low degree of consensus for equine, these five technical solutions were irrelevant for mimicking the migration of plasticizers into human RBCs.

##### Technical Solutions of Natural Origin for Plasma

For plasma, there was a high level of consensus on pig’s plasma: 88% of the experts agreed that this solution was a suitable technical solution for mimicking the migration of plasticizers into human plasma. Bovine plasma also led to a consensus, albeit at a lower level, since 65% of the experts considered this technical solution to be relevant. Canine, ovine, and goat plasmas had a low degree of consensus and rabbit plasma had a moderate degree of consensus with regard to mimicking the migration of plasticizers into human plasma. No consensus was observed for equine plasma because the experts’ opinions were divided.

##### Technical Solutions of Natural Origin for Platelet Concentrate

A moderate degree of consensus was found for pig PC since 81% of the experts agreed that it was a relevant technical solution for mimicking the migration of plasticizers into human PC. No consensus was observed for bovine PC because the experts’ opinions were divided (between 35% and 65%). Canine, equine, ovine, goat, and rabbit PCs had a low or a moderate degree of consensus and were found to be irrelevant for mimicking the migration of plasticizers into human PC.

##### Summary of the Results for Simulants of Natural Origin

The relevance profiles were quite similar for the four LBPs. The majority of the experts were in favour of using pig LBPs for mimicking the migration of plasticizers into human LBPs. Indeed, the experts mentioned that pig’s blood was already used in several types of studies (e.g., in haematology and cardiology) because of its physical–chemical properties (notably the red blood cell size), its stability, and the immunologic similarity between pigs and humans. Bovine WB and plasma were also cited as relevant mimics of human WB and plasma. However, a consensus was not obtained for bovine WB because the red blood cell size stays too different from that of humans compared to the pig’s one. The other technical solutions were not considered to be relevant for simulating the migration of plasticizers into LBPs. Furthermore, the experts emphasized the ease of supply for pig and bovine blood relative to other animal blood (canine or goat blood, for example), for which regulations on animal experiments may be stricter. The blood volume variable was also cited since it was preferable to use blood from an animal with a relatively large volume of blood. In particular, the use of rabbit blood was not recommended since the rather low volume might increase the risk of hemolysis and therefore influence the quality of the blood during sampling. It was mentioned that the use of mammalian plasma would be adequate for mimicking the migration of plasticizers into human plasma. The use of animal models with a fairly high platelet count is required to simulate the migration of plasticizers into human PC: the equine model was not thought to be a good candidate because its platelet count is very low.

#### 3.3.2. Technical Solutions of Synthetic Origin

##### Technical Solutions of Synthetic Origin for Whole Blood

Eight points of consensus were reached for synthetic technical solutions. A high degree of consensus was found for the use of rheological property modifiers and trace elements, since respectively 90% and 85% of the experts agreed that these were suitable technical solutions for preparing a simulant that mimicked the migration of plasticizers into human WB. The use of isotonic solutions, surface tension reducing agents, buffer solutions, and organic compounds to formulate a WB simulant attracted a moderate level of consensus since 80% of the experts considered these technical solutions to be relevant. A lower consensus (70%) was found for the use of a lipid emulsion. With a very low score (30%), a water-soluble dye was found to be irrelevant for a synthetic simulant. It should be noted that no consensus was reached on the other technical solutions (with scores between 35% and 65%).

##### Technical Solutions of Synthetic Origin for Red Blood Cell Concentrate

Five points of consensus were obtained for RBC. A moderate level of consensus was found for rheological property modifiers since 75% of the experts agreed that the use of this type of agent was suitable for mimicking the migration of plasticizers into human RBC. Isotonic solutions and surface tension reducing agents attracted a lower level of consensus since 65% of the experts considered these technical solutions to be relevant. The use of desorbing agents and a water-soluble dye in a synthetic simulant was judged to be inappropriate, and the level of consensus was moderate (25% and 20%, respectively). The experts’ opinions were divided with regard to the other technical solutions, which demonstrated a lack of consensus.

##### Technical Solutions of Synthetic Origin for Plasma

Eleven points of consensus were obtained for plasma. A high degree of consensus was found for the use of buffer solutions because 90% of the experts agreed that these were relevant technical solutions for mimicking the migration of plasticizers into human plasma. A synthetic simulant supplemented with an isotonic solution, a lipid emulsion, a rheological property modifier, a surface tension reducing agent, and certain organic compounds attracted a moderate degree of consensus for relevance. With scores ranging from 15 to 35%, the four other points of consensus attested to the irrelevance of desorbing agents, insoluble particles, preservatives, and water-soluble dyes as components of synthetic simulants for mimicking the migration of plasticizers into human plasma. However, no consensus was observed on the use of stabilizing agents since the experts’ opinions were divided.

##### Technical Solutions of Synthetic Origin for Platelet Concentrate

For PC, six points of consensus were reached. A moderate level of consensus was highlighted for the use of rheology modifying agents and buffer solutions, where respectively 76% and 82% of the experts agreed on the suitability of these technical solutions for mimicking the migration of plasticizers into human PC. The use of an isotonic solution and trace elements led to a lower consensus since, respectively, 71% and 65% of the experts considered these technical solutions to be relevant. The two other points of consensus emphasized the irrelevance of the use of desorbing agents and soluble dyes in an aqueous phase for mimicking the migration of plasticizers into human PC; these technical solutions had scores of 35% and 24%, respectively. However, no consensus was observed for the other technical solutions since the experts’ opinions were divided.

##### Summary of the Results for Simulants of Synthetic Origin

In contrast to the results for the simulants of natural origin, profiles for the four LBPs differed when considering the simulants of synthetic origin. However, it was noted that the use of an isotonic solution and a rheological property modifier might be of value in a simulant capable of mimicking the migration of plasticizers from a PVC bag into an LBP. The other technical solutions for simulants must be studied specifically for each LBP. The experts differed in their estimation of the value of synthetic simulants. Some experts considered that it is essential to use natural blood components, while others considered that simulation of the affinity between plasticizers and the LBP is most important—even if the simulant’s chemical composition does not match that of the LBP. Hence, the use of synthetic technical solutions appears to be relevant as long as the proportions of each of the constituents are considered carefully. For the development of simulants, particular attention was paid to the following parameters: pH, viscosity, osmolarity, ionic strength, hydrophobicity, and density. The notion of the complexity of a mixture was also raised by several experts. Indeed, it was often stated that only the components necessary for the development of plasticizer migration simulants should be used, i.e., so that the technical solution is as simple as possible. For example, the use of insoluble particles, water-soluble dyes, preservatives, and stabilizers was widely considered to be non-essential. Moreover, it was suggested that non-essential components might interfere with the essential components and might thus impair the simulant’s ability to mimic the migration of plasticizers into the LBP.

## 4. Conclusions

Our Delphi working group was able to set out specifications for the development of human LBP simulants and evaluated technical solutions that might meet these specifications. Despite the Delphi method’s inherent limitations and the absence of a truly international context (our study was limited to France and Belgium), the study’s main strength was the multidisciplinary working group on the development of simulants. In contrast to most usual Delphi studies, we decided not to carry out a second survey after the initial questionnaires had been processed. Indeed, the proposals made by the experts were very precise and were easily integrated into the categories studied here. The comparison of a number of relevant natural or synthetic products with human blood is now warranted, with a view to assessing their experimental relevance and determining the amount of plasticizer that transfers from PVC medical devices to LBPs.

The points of consensus highlighted by our qualitative study might constitute a database for a draft approach to the standardization of plasticizer migration tests, in which human blood would be replaced by a natural or synthetic simulant. Among all proposed technical solutions, pig’s blood could be the most suitable and convenient one. Indeed, pig’s blood possesses the closest physical–chemical and biological properties to human blood, and it can be processed to obtain plasma, PC, and RBC using the same protocol applied for human blood. The availability and standardization of pigs for experimental research also constitute two clear benefits. However, it could be necessary to check that pig’s blood is not contaminated by plasticizers before migration studies.

This approach could also be extended to other types of biological tests in order to reduce the need for scarce human blood.

## Figures and Tables

**Table 1:** Set of keywords (queries) used in the literature search.

List Number	Keywords
1	Containers OR Bags OR Kits OR Pouch OR Packs OR Medical devices OR Medical grade OR Blood bags system
2	Material * OR Phthalate * OR “non-DEHP” OR Plasticiz * OR Di(2-ethylhexyl)phthalate OR (DEHP AND free) OR Polyvinylchloride OR Ester * OR PVC OR Plasticis * OR Bis(2-ethylhexyl) phthalate OR Di(2-ethylhexyl) phthalate OR BIS(2-ETHYLHEXYL)PHTHALATE OR Di-2-ethylhexyl phthalate OR Polyvinyl chloride
3	Blood OR Plasma OR Platelets OR Erythrocytes OR Hematology OR Transfusion OR Thrombocyte OR Intravenous * OR Parenteral *
4	Simulant * OR Mimic *
5	Leach * OR Extract * OR Release * OR Diffus * OR Deliv *

Note: an asterisk (*) denotes words beginning with the indicated root, and quotes denote a search for the exact term.

**Table 2:** Consensus scale for the relevance of identified technical solutions.

Score (%)	Degree of Consensus
x ≥ 85	High degree of relevance
75 ≤ x ≤ 84	Moderate degree of relevance
65 ≤ x ≤ 74	Low degree of relevance
35 < x < 65	Lack of consensus
26 ≤ x ≤ 35	Low degree of irrelevance
16 ≤ x ≤ 25	Moderate degree of irrelevance
x ≤ 15	High degree of irrelevance

**Table 3:** Reformulated criteria suggested by the experts in the nominal group for each LBP.

Criterion	Abbreviation	Labile Blood Product			
		WB	RBC	P	PC
Physical characteristics (viscosity, surface tension, thermolability, and density (centrifugation behaviour))	Physical characteristics 1	√	X	X	X
Physical characteristics (viscosity, surface tension, thermolability, and density)	Physical characteristics 2	X	√	√	√
Chemical characteristics (hydrophilicity, lipophilicity, pH, surface tension, osmolarity, ionic forces, polarity, and strong/weak bond interactions)	Chemical characteristics	√	√	√	√
Product opacity	Opacity	√	√	X	X
Form (size/shape of cells/particles, suspension/solution)	Form	√	√	√	√
Stability during preparation, storage and use (time, temperature, light, pressure, centrifugation, and processing of the blood product)	Stability	√	√	√	√
Organic/inorganic chemical composition (sugar (glucose), proteins (transport proteins and albumin), phospholipids, cell phase/lipid phase ratio, absence of plasticizer, liposomes, bile acids, presence of anticoagulant or preservative solutions in the LBP, and the presence of substances that can clot)	Composition 1	√	√	X	√
Organic/inorganic chemical compositions (sugar (glucose), proteins (transport proteins and albumin), phospholipids, absence of plasticizer, liposomes, bile acids, presence of anticoagulant or preservative solutions in the LBP, and the presence of substances that can clot)	Composition 2	X	X	√	X
Ability to mimic a particular clinical situation (hyperuricaemia, hyperlactatemia, hyperglycemia, acidosis, hemolysis, and the heterogeneity of patients’ blood)	Ability to mimic a particular clinical situation	√	√	√	√
Ease and safety of use: cost, ease of supply, ease of disposal, inter-batch reproducibility, and ability to combine products	Ease and safety of use	√	√	√	√
Blood-correlated simulant-plastic interaction: ability to dissolve in the simulant, and structural change with plasticizers	Simulant-plastic interaction	√	√	√	√
Compatibility with extraction and analysis method	Compatibility	√	√	√	√
Number of criteria		10	10	9	9

WB: whole blood; RBC: red blood cell concentrate; P: plasma; PC: platelet concentrate; √: applicable; X: not applicable.

**Table 4:** Three-level classification of the criteria from the nominal group’s qualitative analysis.

Criteria	LBP			
	WB	RBC	P	PC
Important	“Physical characteristics” “Chemical characteristics” “Composition”			
Moderately important	“Stability” “Ease and safety of use” “Simulant–plastic interaction”			“Form” “Stability” “Ease and safety of use” “Simulant–plastic interaction”
Less important	“Opacity” “Form” “Ability to mimic a particular clinical situation” “Compatibility”		“Form” “Ability to mimic a particular clinical situation” “Compatibility”	“Ability to mimic a particular clinical situation” “Compatibility”

LPB: labile blood product; WB: whole blood; RBC: red blood cell concentrate; P: plasma; PC: platelet concentrate.

**Table 5:** Ranking of the criteria for each labile blood product.

Criterion	WB		RBC		P		PC	
	Ranking	Score	Ranking	Score	Ranking	Score	Ranking	Score
Chemical characteristics	1	32	1	33	1	29	1	30
Physical characteristics	2	37	2	34	2	30	2	32
Compositions	3	39	3	40	3	42	3	42
Stability	4	59	4	51	4	61	4	54
Simulant–plastic interaction	5	64	5	66	5	63	5	67
Ease and safety of use	6	69	6	70	6	70	6	71
Forms	7	81	7	77	8	90	7	72
Compatibility	8	82	8	79	7	77	8	81
Ability to mimic a particular clinical situation	9	104	9	98	9	97	9	91
Opacity	10	109	10	104	-	-	-	-

WB: whole blood; RBC: red blood cell concentrate; P: plasma; PC: platelet concentrate; -: no value.

**Table 6:** Classification of technical solutions.

Technical Solution		References
Origin	Suggestion	
Natural	Pig’s blood	[7,8,9,10,11,12,13,14,15,16,17,18,19,20,21,22,23,24,25,26]
	Bovine blood	[8,12,19,22,24,27,28,29,30,31,32,33,34]
	Canine blood	[35,36,37]
	Equine blood	[8]
	Ovine blood	[8]
	Goat blood	[37]
	Rabbit blood	[38]
Synthetic	Isotonic solutions: physiological saline solutions, NaCl 0.9%, etc.	[8,9,12,15,19,21,25,27,34,39,40,41,42,43,44,45]
	Lipid emulsions: parenteral nutrition products, etc.	[39,46,47,48,49]
	Rheological property modifiers: glycerol, gelatine, gellan gum, cellulose and its derivatives, dextran, albumin, silicone oil, polyacrylamide, cutting fluid, etc.	[9,12,13,26,27,30,40,41,42,43,44,45,50,51,52,53,54,55,56,57,58,59,60,61,62,63,64,65,66,67,68,69,70,71,72,73,74,75,76,77,78,79,80,81,82,83,84,85,86]
	Desorption agents: solvents, silicone oil, etc.	[45,87]
	Insoluble particles: carbon fibres, nylon, polyethene and polystyrene microspheres, polyamide particles, silicon carbides, etc.	[9,12,53,55,56,58,59,62,76,78,81,82,83,84,88,89]
	Surface tension reducing agents: alcohols, glycoproteins, fatty acids, etc.	[45,87]
	Preservatives: copper sulfate, potassium sorbate, antifungal agents, sodium azide, etc.	[59,73]
	Stabilizing agents: starch, xanthan gum, gellan gum, surfactants like lecithin, etc.	[30,45,58,62,63,64,84,85]
	Buffer solutions: potassium phosphate, etc.	[45,86]
	Trace elements: potassium chloride, sodium iodide, magnesium sulfate, calcium gluceptate, etc.	[39,72]
	Organic compounds: amino acids, dextrose, etc.	[9,13,27,39]
	Water-soluble dyes: beetroot juice, etc.	[45]

## Data Availability

The data presented in this study are available on request from the corresponding author. The data are not publicly available due to privacy.

## Supplementary Materials

The following are available online at https://www.mdpi.com/article/10.3390/biom11081081/s1, Table S1: Experts’ degree, function, field of expertise and experience.

## References

[1] Fernandez-Canal C., Pinta P.-G., Eljezi T., Larbre V., Kauffmann S., Camilleri L., Cosserant B., Bernard L., Pereira B., Constantin J.-M. (2018). Patients’ Exposure to PVC Plasticizers from ECMO Circuits. Expert. Rev. Med. Devices..

[2] Prowse C.V., de Korte D., Hess J.R., van der Meer P.F., Biomedical Excellence for Safer Transfusion (BEST) Collaborative (2014). Commercially Available Blood Storage Containers. Vox Sang..

[3] Hsu C.-C., Sandford B. (2019). The Delphi Technique: Making Sense of Consensus. Pract. Assess. Res. Eval..

[4] Fink A., Kosecoff J., Chassin M., Brook R.H. (1984). Consensus Methods: Characteristics and Guidelines for Use. Am. J. Public Health.

[5] Jones J., Hunter D. (1995). Consensus Methods for Medical and Health Services Research. BMJ.

[6] Visade F., Lefebvre A., Floret E., Decaudin B., Puisieux F., Delecluse C., Beuscart J.-B. (2019). Proposition of a structured list of information items to be transmitted to primary caregivers after in-hospital medication optimization: A qualitative study. Acta Clin. Belg..

[7] Mercado-Shekhar K.P., Su H., Kalaikadal D.S., Lorenz J.N., Manglik R.M., Holland C.K., Redington A.N., Haworth K.J. (2019). Acoustic Droplet Vaporization-Mediated Dissolved Oxygen Scavenging in Blood-Mimicking Fluids, Plasma, and Blood. Ultrason. Sonochem..

[8] Weng X., Cloutier G., Pibarot P., Durand L.G. (1996). Comparison and Simulation of Different Levels of Erythrocyte Aggregation with Pig, Horse, Sheep, Calf, and Normal Human Blood. Biorheology.

[9] Cloutier G., Shung K.K. Cyclic Variation of Doppler Backscattering Power from Porcine Blood in a Pulsatile Flow Model. Proceedings of the IEEE 1991 Ultrasonics Symposium.

[10] Wu S.J., Shung K.K. (1996). Cyclic Variation of Doppler Power from Whole Blood under Pulsatile Flow. Ultrasound Med. Biol..

[11] Brookshier K.K., Tarbell J.M. (1991). Effect of Hematocrit on Wall Shear Rate in Oscillatory Flow: Do the Elastic Properties of Blood Play a Role?. Biorheology.

[12] Allard L., Cloutier G., Durand L. (1996). Effect of the Insonification Angle on the Doppler Backscattered Power under Red Blood Cell Aggregation Conditions. IEEE T. Ultrason. Ferr..

[13] Cloutier G., Shung K.K., Durand L.G. (1993). Experimental Evaluation of Intrinsic and Nonstationary Ultrasonic Doppler Spectral Broadening in Steady and Pulsatile Flow Loop Models. IEEE Trans. Ultrason. Ferr..

[14] Badimon L., Padro T., Vilahur G. (2012). Extracorporeal Assays of Thrombosis. Methods Mol. Biol..

[15] Kuo I.Y., Shung K.K. (1994). High Frequency Ultrasonic Backscatter from Erythrocyte Suspension. IEEE Trans. Biomed. Eng..

[16] Badimon J.J., Lettino M., Toschi V., Fuster V., Berrozpe M., Chesebro J.H., Badimon L. (1999). Local Inhibition of Tissue Factor Reduces the Thrombogenicity of Disrupted Human Atherosclerotic Plaques: Effects of Tissue Factor Pathway Inhibitor on Plaque Thrombogenicity under Flow Conditions. Circulation.

[17] Mo L.Y., Yip G., Cobbold R.S., Gutt C., Joy M., Santyr G., Shung K.K. (1991). Non-Newtonian Behavior of Whole Blood in a Large Diameter Tube. Biorheology.

[18] Greaby R., Zderic V., Vaezy S. (2007). Pulsatile Flow Phantom for Ultrasound Image-Guided HIFU Treatment of Vascular Injuries. Ultrasound Med. Biol..

[19] Cloutier G., Qin Z., Lees S., Ferrari L.A. (1997). Shear Rate Dependence of Normal, Hypo-, and Hyper-Aggregating Erythrocytes Studied with Power Doppler Ultrasound. Acoustical Imaging.

[20] Cloutier G., Shung K.K. (1993). Study of Red Cell Aggregation in Pulsatile Flow from Ultrasonic Doppler Power Measurements. Biorheology.

[21] Cloutier G., Shung K.K. The Effect of Turbulence on the Variation of the Ultrasonic Doppler Amplitude within the Cardiac Cycle. Proceedings of the 14th Annual International Conference of the IEEE Engineering in Medicine and Biology Society.

[22] Shung K.K., Cloutier G., Lim C.C. (1992). The Effects of Hematocrit, Shear Rate, and Turbulence on Ultrasonic Doppler Spectrum from Blood. IEEE Trans. Biomed. Eng..

[23] Yuan Y.W., Shung K.K. (1988). Ultrasonic Backscatter from Flowing Whole Blood. I: Dependence on Shear Rate and Hematocrit. J. Acoust. Soc. Am..

[24] Yuan Y.W., Shung K.K. (1988). Ultrasonic Backscatter from Flowing Whole Blood. II: Dependence on Frequency and Fibrinogen Concentration. J. Acoust. Soc. Am..

[25] Mo L.Y., Kuo I.Y., Shung K.K., Ceresne L., Cobbold R.S. (1994). Ultrasound Scattering from Blood with Hematocrits up to 100%. IEEE Trans. Biomed. Eng..

[26] Embree P.M., O’Brien W.R. (1990). Volumetric Blood Flow via Time-Domain Correlation: Experimental Verification. IEEE T. Ultrason. Ferr..

[27] Cloutier G., Allard L., Durand L.G. (1995). Changes in Ultrasonic Doppler Backscattered Power Downstream of Concentric and Eccentric Stenoses under Pulsatile Flow. Ultrasound Med. Biol..

[28] Cloutier G., Allard L., Durand L.G. (1996). Characterization of Blood Flow Turbulence with Pulsed-Wave and Power Doppler Ultrasound Imaging. J. Biomech. Eng..

[29] Shung K.K., Yuan Y.W., Fei D.Y., Tarbell J.M. (1984). Effect of Flow Disturbance on Ultrasonic Backscatter from Blood. J. Acoust. Soc. Am..

[30] Mann D.E., Tarbell J.M. (1990). Flow of Non-Newtonian Blood Analog Fluids in Rigid Curved and Straight Artery Models. Biorheology.

[31] Yuan Y.W., Shung K.K. (1984). Further Studies on Ultrasonic Backscatter from Blood. Ultrason. Symp..

[32] Lucas R.J., Twersky V. (1987). Inversion of Ultrasonic Scattering Data for Red Blood Cell Suspensions under Different Flow Conditions. J. Acoust. Soc. Am..

[33] Berger N.E., Lucas R.J., Twersky V. (1991). Polydisperse Scattering Theory and Comparisons with Data for Red Blood Cells. J. Acoust. Soc. Am..

[34] Fayz S., Herbert R., Martin A.M. (1977). The Release of Plasticizer from Polyvinyl Chloride Haemodialysis Tubing. J. Pharm. Pharmacol..

[35] Kripfgans O.D., Fowlkes J.B., Miller D.L., Eldevik O.P., Carson P.L. (2000). Acoustic Droplet Vaporization for Therapeutic and Diagnostic Applications. Ultrasound Med. Biol..

[36] Yamada E.G., Fitzgerald P.J., Sudhir K., Hargrave V.K., Yock P.G. (1992). Intravascular Ultrasound Imaging of Blood: The Effect of Hematocrit and Flow on Backscatter. J. Am. Soc. Echocardiogr..

[37] Borders S.E., Fronek A., Kemper W.S., Franklin D. (1978). Ultrasonic Energy Backscattered from Blood. An Experimental Determination of the Variation of Sound Energy with Hematocrit. Ann. Biomed. Eng..

[38] Pajek D., Burgess A., Huang Y., Hynynen K. (2014). High-Intensity Focused Ultrasound Sonothrombolysis: The Use of Perfluorocarbon Droplets to Achieve Clot Lysis at Reduced Acoustic Power. Ultrasound Med. Biol..

[39] Mazur H.I., Stennett D.J., Egging P.K. (1989). Extraction of Diethylhexylphthalate from Total Nutrient Solution-Containing Polyvinyl Chloride Bags. J. Parenter. Enter. Nutr..

[40] Nandy S., Tarbell J.M. (1986). Flush-Mounted Hot Film Anemometer Accuracy in Pulsatile Flow. J. Biomech. Eng..

[41] Yongchareon W., Young D.F. (1979). Initiation of Turbulence in Models of Arterial Stenoses. J. Biomech..

[42] Landwehr P., Schindler R., Heinrich U., Dölken W., Krahe T., Lackner K. (1991). Quantification of Vascular Stenosis with Color Doppler Flow Imaging: In Vitro Investigations. Radiology.

[43] Chahed N., Péronneau P., Delouche A., Diebold B. (1991). Velocity Profiles and Streamlines of a Revolution Post-Stenotic Flow. Biorheology.

[44] Walburn F.J., Stein P.D. (1981). Velocity Profiles in Symmetrically Branched Tubes Simulating the Aortic Bifurcation. J. Biomech..

[45] McIlhenny S.E. (2014). Blood and Bone Marrow Simulant.

[46] Loff S., Hannmann T., Subotic U., Reinecke F.-M., Wischmann H., Brade J. (2008). Extraction of Diethylhexylphthalate by Home Total Parenteral Nutrition from Polyvinyl Chloride Infusion Lines Commonly Used in the Home. J. Pediatr. Gastroenterol. Nutr..

[47] Loff S., Kabs F., Subotic U., Schaible T., Reinecke F., Langbein M. (2002). Kinetics of Diethylhexyl-Phthalate Extraction From Polyvinylchloride-Infusion Lines. J. Parenter. Enteral. Nutr..

[48] Faessler D., McCombie G., Biedermann M., Felder F., Subotic U. (2017). Leaching of Plasticizers from Polyvinylchloride Perfusion Lines by Different Lipid Emulsions for Premature Infants under Clinical Conditions. Int. J. Pharm..

[49] Loff S., Kabs F., Witt K., Sartoris J., Mandl B., Niessen K.H., Waag K.L. (2000). Polyvinylchloride Infusion Lines Expose Infants to Large Amounts of Toxic Plasticizers. J. Pediatr. Surg..

[50] Hoskins P.R., Loupas T., McDicken W.N. (1990). A Comparison of the Doppler Spectra from Human Blood and Artificial Blood Used in a Flow Phantom. Ultrasound Med. Biol..

[51] Hoskins P.R., Anderson T., McDicken W.N. (1989). A Computer Controlled Flow Phantom for Generation of Physiological Doppler Waveforms. Phys. Med. Biol..

[52] Hein I.A., O’Brien W.D. (1992). A Flexible Blood Flow Phantom Capable of Independently Producing Constant and Pulsatile Flow with a Predictable Spatial Flow Profile for Ultrasound Flow Measurement Validations. IEEE Trans. Biomed. Eng..

[53] Dabrowski W., Dunmore-Buyze J., Cardinal H.N., Fenster A. (2001). A Real Vessel Phantom for Flow Imaging: 3-D Doppler Ultrasound of Steady Flow. Ultrasound Med. Biol..

[54] McDicken W.N. (1986). A Versatile Test-Object for the Calibration of Ultrasonic Doppler Flow Instruments. Ultrasound Med. Biol..

[55] Rickey D.W., Picot P.A., Christopher D.A., Fenster A. (1995). A Wall-Less Vessel Phantom for Doppler Ultrasound Studies. Ultrasound Med. Biol..

[56] Samavat H., Evans J.A. (2006). An Ideal Blood Mimicking Fluid for Doppler Ultrasound Phantoms. J. Med. Phys..

[57] Douville Y., Johnston K.W., Kassam M., Zuech P., Cobbold R.S., Jares A. (1983). An in Vitro Model and Its Application for the Study of Carotid Doppler Spectral Broadening. Ultrasound Med. Biol..

[58] Poepping T.L., Nikolov H.N., Rankin R.N., Lee M., Holdsworth D.W. (2002). An in Vitro System for Doppler Ultrasound Flow Studies in the Stenosed Carotid Artery Bifurcation. Ultrasound Med. Biol..

[59] Lubbers J. (1999). Application of a New Blood-Mimicking Fluid in a Flow Doppler Test Object. Eur. J. Ultrasound.

[60] Khalifa A.M., Giddens D.P. (1981). Characterization and Evolution Poststenotic Flow Disturbances. J. Biomech..

[61] Winkler A.J., Wu J. (1995). Correction of Intrinsic Spectral Broadening Errors in Doppler Peak Velocity Measurements Made with Phased Sector and Linear Array Transducers. Ultrasound Med. Biol..

[62] Liu Y., Maruvada S., King R.L., Herman B.A., Wear K.A. (2008). Development and Characterization of a Blood Mimicking Fluid for High Intensity Focused Ultrasound. J. Acoust. Soc. Am..

[63] Teirlinck C.J., Bezemer R.A., Kollmann C., Lubbers J., Hoskins P.R., Ramnarine K.V., Fish P., Fredeldt K.E., Schaarschmidt U.G. (1998). Development of an Example Flow Test Object and Comparison of Five of These Test Objects, Constructed in Various Laboratories. Ultrasonics.

[64] Ramnarine K.V., Hoskins P.R., Routh H.F., Davidson F. (1999). Doppler Backscatter Properties of a Blood-Mimicking Fluid for Doppler Performance Assessment. Ultrasound Med. Biol..

[65] Hutchison K.J. (1993). Doppler Ultrasound Spectral Shape in the Poststenotic Velocity Field. Ultrasound Med. Biol..

[66] Seeley B.D., Young D.F. (1976). Effect of Geometry on Pressure Losses across Models of Arterial Stenoses. J. Biomech..

[67] Solzbach U., Wollschläger H., Zeiher A., Just H. (1987). Effect of Stenotic Geometry on Flow Behaviour across Stenotic Models. Med. Biol. Eng. Comput..

[68] Fei D.Y., Billian C., Rittgers S.E. (1988). Flow Dynamics in a Stenosed Carotid Bifurcation Model--Part I: Basic Velocity Measurements. Ultrasound Med. Biol..

[69] Tortoli P., Thompson R.S., Berti P., Guidi F. (1999). Flow Imaging with Pulsed Doppler Ultrasound and Flow Phantoms. IEEE Trans. Ultrason. Ferr..

[70] Moravec S., Liepsch D. (1983). Flow Investigations in a Model of a Three-Dimensional Human Artery with Newtonian and Non-Newtonian Fluids. Part, I. Biorheology.

[71] Tortoli P., Guidi G., Newhouse V.L. (1995). Improved Blood Velocity Estimation Using the Maximum Doppler Frequency. Ultrasound Med. Biol..

[72] Hariharan P., Aycock K.I., Buesen M., Day S.W., Good B.C., Herbertson L.H., Steinseifer U., Manning K.B., Craven B.A., Malinauskas R.A. (2018). Inter-Laboratory Characterization of the Velocity Field in the FDA Blood Pump Model Using Particle Image Velocimetry (PIV). Cardiovasc. Eng. Technol..

[73] Feinberg D.A., Crooks L.E., Sheldon P., Hoenninger J., Watts J., Arakawa M. (1985). Magnetic Resonance Imaging the Velocity Vector Components of Fluid Flow. Magn. Reson. Med..

[74] Kraft K.A., Fatouros P.P., Fei D.Y., Rittgers S.E., Kishore P.R. (1989). MR Imaging of Model Fluid Velocity Profiles. Magn Reson Imaging.

[75] Boote E.J., Zagzebski J.A. (1988). Performance Tests of Doppler Ultrasound Equipment with a Tissue and Blood-Mimicking Phantom. J. Ultrasound Med..

[76] Liepsch D., Moravec S. (1984). Pulsatile Flow of Non-Newtonian Fluid in Distensible Models of Human Arteries. Biorheology.

[77] Brown T.D., Gabel R.H., Pedersen D.R., Bell L.D., Blair W.F. (1985). Some Characteristics of Laminar Flow Velocity Spectra Detected by a 20 MHz Pulsed Ultrasound Doppler. J. Biomech..

[78] Steinman A.H., Tavakkoli J., Myers J.G., Cobbold R.S., Johnston K.W. (2001). Sources of Error in Maximum Velocity Estimation Using Linear Phased-Array Doppler Systems with Steady Flow. Ultrasound Med. Biol..

[79] Siouffi M., Pelissier R., Farahifar D., Rieu R. (1984). The Effect of Unsteadiness on the Flow through Stenoses and Bifurcations. J. Biomech..

[80] Ku D.N., Liepsch D. (1986). The Effects of Non-Newtonian Viscoelasticity and Wall Elasticity on Flow at a 90 Degrees Bifurcation. Biorheology.

[81] Guo Z., Fenster A. (1996). Three-Dimensional Power Doppler Imaging: A Phantom Study to Quantify Vessel Stenosis. Ultrasound Med. Biol..

[82] Oates C.P. (1991). Towards an Ideal Blood Analogue for Doppler Ultrasound Phantoms. Phys. Med. Biol..

[83] Giddens D.P., Khalifa A.M. (1982). Turbulence Measurements with Pulsed Doppler Ultrasound Employing a Frequency Tracking Method. Ultrasound Med. Biol..

[84] Ramnarine K.V., Nassiri D.K., Hoskins P.R., Lubbers J. (1998). Validation of a New Blood-Mimicking Fluid for Use in Doppler Flow Test Objects. Ultrasound Med. Biol..

[85] Ahmed S.A., Giddens D.P. (1983). Velocity Measurements in Steady Flow through Axisymmetric Stenoses at Moderate Reynolds Numbers. J. Biomech..

[86] Hood W.H., Spears M.J., Worley W.J. (2010). Menstrual Fluid Simulant Containing Blood Product, Gelatin, Polyacrylamide and Buffer.

[87] Bernard L., Cueff R., Breysse C., Décaudin B., Sautou V., Armed Study Group (2015). Migrability of PVC Plasticizers from Medical Devices into a Simulant of Infused Solutions. Int. J. Pharm..

[88] Yuan D., Zhang J., Sluyter R., Zhao Q., Yan S., Alici G., Li W. (2016). Continuous Plasma Extraction under Viscoelastic Fluid in a Straight Channel with Asymmetrical Expansion-Contraction Cavity Arrays. Lab Chip.

[89] Cloutier G., Shung K.K. (1993). Cyclic Variation of the Power of Ultrasonic Doppler Signals Backscattered by Polystyrene Microspheres and Porcine Erythrocyte Suspensions. IEEE Trans. Biomed. Eng..

