# Supplementary material for: Specification and Evaluation of Plasticizer Migration Simulants for Human Blood Products: A Delphi Study
Source: Biomolecules. 2021 Jul 22;11(8):1081. doi: 10.3390/biom11081081 (PMC8392596; doi:10.3390/biom11081081)
Supplement: Supplementary file 1 [file biomolecules-11-01081-s001.zip › biomolecules-1282781-supplementary.pdf]

biomolecules MDPI

Supplementary information

Table S1. Experts' degree, function, field of expertise and experience

Expert's Name	Degree	Function	Field of Expertise	Experience
S Chatellier	PhD	Chief Executive Officer	Biology and transfusion medical devices	> 25 years
F. Cognasse	PhD	Research Director and scientific Director	Biology	> 15 years
C. Danel	PhD	Assistant professor	Analytical chemistry	> 15 years
B. Delorme	PhD	Scientific manager - R&D department	Biotherapy medical devices	> 25 years
L. Ducoroy	PhD	Engineer R&D	Materials sciences	> 15 years
A. Dupont	PhD, PharmD	Professor and hospital practitioner	Clinical pharmacy	> 20 years
G. Hénard	Engineer	Components division manager - R&D department	Transfusion medical devices	> 25 years
D. Garrigue	MD	Intensive care anaesthetist	Anesthesia	> 20 years
S. Genay	PhD, PharmD	Assistant professor and hospital practitioner	Clinical pharmacy	> 10 years
JF. Goossens	PhD	Professor	Analytical chemistry	> 25 years
L. Goossens	PhD	Assistant professor	Organic chemistry	> 20 years
C. Havez	Engineer	Engineer R&D	Biology and transfusion medical devices	> 5 years
JD. Hecq	PhD, PharmD	Professor and hospital practitioner	Galenic pharmacy	> 30 years
M. Jeanne	PhD, MD	Intensive care anaesthetist	Anesthesia	> 15 years
M. Lecoeur	PhD	Assistant professor	Analytical chemistry	> 10 years
L. Leroy	PhD	Engineer R&D	Materials sciences	> 5 years
C. Maeght	Engineer	Engineer R&D	Materials sciences	> 10 years
I. Mendel	MD	Hemovigilance and transfusion safety manager	Hematology	> 25 years
T. Najdovski	PhD	Labile Blood Products Production Manager	Chemistry and biology	> 30 years
P. Odou	PhD	Professor and hospital practitioner	Galenic pharmacy	> 25 years
M. Pierre	Master	Engineer R&D	Analytical chemistry	> 5 years
A. Rapaille	Master	Quality control manager	Biology	> 40 years
G. Saint-Lorant	PhD, PharmD	Assistant professor and hospital practitioner	Clinical pharmacy	> 10 years
A. Ung	Master	Engineer	Biology and hematology	> 15 years
